# Assessment of electronic health literacy and its associated factors among internet -using primary health care attendees in Mansoura district, Egypt: a cross-sectional study

**DOI:** 10.1186/s12889-026-28907-8

**Published:** 2026-08-19

**Authors:** Shymaa Mamdouh Mohamed Abdu, Sahar Mohamed Youness, Abdel-Hady El-Gilany

**Affiliations:** 1 Public health and Community medicine Department, Mansoura University, Faculty ofMedicine, Mansoura City, Egypt; 2 Public health & Preventive medicine, Egypt Ministry of Health and Population, Mansoura City, Egypt

**Keywords:** Internet use, e Health literacy, Barriers, Primary health care

## Abstract

**Background:**

People need electronic health literacy (eHealth literacy) to find and assess reliable health information. Little research has been conducted on eHealth literacy in low- and middle-income countries (LMICs) including Egypt compared to developed countries. This study aimed to assess e Health literacy level, its associated factors and explored perceived barriers among internet using primary health care (PHC) attendees.

**Methods:**

A cross-sectional analytical study was carried out among 532 adult internet using PHC attendees selected by systematic random sampling technique. A structured valid Arabic questionnaire was used including data about socio-demographic and internet use characteristics, Arabic ehealth literacy Scale (Ar-eHEALS), and barriers of e Health literacy. Multiple logistic regression analysis was used to detect the significant factors of e Health literacy.

**Results:**

The mean age of study participants was 24.6 ± 8.9. The majority of them were females (75%). More than two-thirds (67.3%) of participants experienced adequate e Health literacy, while 32.7% had limited one. Female sex (AOR = 1.8, 95% CI (1.1-3), perceived internet usefulness (AOR = 5.6, 95% CI (2.9–10.7), perceived internet importance (AOR = 5, 95% CI (1.8–13.3), better self –rated health status (AOR = 3.7, 95% CI (1.6–8.9), and having higher general health knowledge (AOR = 2.9, 95% CI (1.4–5.9) were independently associated factors of adequate e Health literacy. Low basic literacy, limited technology skills, lack of health care providers’ interaction, and cultural aspects perceived as the most frequently barriers.

**Conclusion:**

Despite a significant percentage of participants having adequate e Health literacy, there are still considerable barriers perceived among participants. Improving technology skills, empowering person-provider interaction, and providing culturally sensitive electronic health means are vital to enhance e Health literacy and reduce disparities to access a reliable online health content. The non-representative sample limit the generalizability to all PHC attendees.

**Supplementary Information:**

The online version contains supplementary material available at 10.1186/s12889-026-28907-8.

## Introduction

Information communication technology (ICT) progresses have promoted numerous sectors, including health. Electronic health (E health) refers to the use of ICT to support health care, surveillance, literature, and education. E health is one of the fastest-growing health fields [1]. The World Health Organization (WHO) also recognized the potential of E health to support the Sustainable Development Goals (SDGs) by improving health service quality, accessibility, and affordability in all countries [2]. Globally, the internet has become the primary source of health information. People who have access to information online are more likely to have better health literacy [3].This change was also observed in LMICs, including Egypt [4, 5].

E health literacy is the basic ability of people to seek out, comprehend and utilize digital health information and apply it to a health issue [6].The confluence point between e-health and traditional health literacy [7]. In today’s digital age, the ability to access, understand and use health information has also become vital for both patients and health care providers [8]. In addition, understanding the level of eHealth literacy is essential in developing public health campaigns that are inclusive by design, practical, and effective [9].

E health literacy is based on traditional health literacy and includes information, media, health and computer skills (LILY model) [10]. Healthcare professionals should assess patients’ e Health literacy before providing digital health tools to guarantee informed and successful utilization [11]. E health literacy can lead to higher patient satisfaction, which is a key determinant of treatment adherence. Digital health services can facilitate more meaningful relations between physicians and patients with chronic diseases [12]. Therefore, e Health literacy is affected by various socio-demographic characteristics that include age, gender, education, and socioeconomic level [10]. Moreover, Alhuwail and Abdulsalam [13] stated that people’s knowledge of technology, the internet, and other online sources of health information are strong independent factors of their e Health literacy.

The digital divide is the difference between people with access to, use of and profit from digital technologies. This notion may be separated into 3 aspects based on 3-stage digital divide, which are digital access divide, digital use divide and digital outcome divide. The accessibility divide is caused by poor internet connectivity, unavailability of computing devices, software and peripherals and lack of interest in utilizing the internet [14]. Technology access is a major issue for rural, low-income, and elderly individuals [15]. Considerable investment and a proliferation of digital health tools (DHTs) are available to health care consumers; yet, consumption among specific population groups has been limited [16].Thus, DHTs could exacerbate the current inequities in our health care system by establishing a digital divide that impacts the effective and equitable delivery of care [17]. This reduced uptake of DHTs may be partly because the cultural, language, and health literacy needs of various populations were not considered. When designing DHTs, it is vital to understand the viewpoints of the end users themselves (e.g., customers, patients, and carers) on the aspects that influence their use of digital health [18].

Following the last COVID-19 pandemic, Egypt has announced its National Digital Health Policy for 2025–2029, a comprehensive plan to accelerate the digital transformation of the nation’s healthcare system [19]. The growth in the number of Internet users, the need for health information, and the use of smartphones indicate the importance of the assessment of e Health literacy. Moreover, improved e Health literacy enables individuals to be more proactive about their health, including seeking preventive screenings [20].

Most studies on eHealth literacy were carried out mostly in developed and high-income countries [21–23] and in the Arab countries including Kuwait [13], Jordan [24], and Lebanon [9]. But, within PHC context, few studies assessed the level of e Health literacy [25–27]. Moreover, few Egyptian studies assessed e Health literacy. A recent Egyptian research was conducted among humanistic science students at Tanta University and found that 44% of them had limited digital health literacy [28]. Another study conducted among medical students at Helwan University found that 87.1% of them searched for health information online, yet many were unaware of its credibility [29]. Such findings focused on students, and the level of e Health literacy among the public remains inadequately discovered in Egypt. Moreover, e Health literacy can only be meaningfully assessed among people who have access to and use the internet. To our knowledge, there are no prior studies assessed the level of e Health literacy and its associated factors among internet using PHC attendees in Egypt. Addressing this gap is key to developing targeted interventions that improve equitable access to digital health information and minimize health disparities. Therefore, this study aimed to assess the level of e Health literacy and its associated factors, and to explore the perceived barriers among internet using PHC attendees in Mansoura district, Egypt.

## Methods

### Study design

A facility based- cross- sectional analytical study was carried out over two consecutive months (from 21 February to 30 April 2026).

### Setting

This study was conducted in PHC facilities (rural and urban) of Mansoura District, Dakahlia governorate, Egypt. Dakahlia Governorate is located in the northeastern part of the Arab Republic of Egypt, within the Nile Delta region. It is one of the most important governorates of Lower Egypt in terms of population density and agricultural and industrial activity. Mansoura city is the capital of Dakahlia governorate and one of its 18 districts. Mansoura Health District includes 10 family medicine centers, 31 family medicine units, 1 outpatient clinic, 4 health offices, 6 district health units (wehda ahyayiya) units, and 2 maternity and childhood care centers. Moreover, the Mansoura Heath District includes both rural and urban PHC settings which allows recruiting participants from various socio-demographic backgrounds.

### Target population

 PHC attendees who are current internet users, aged 18 years and more and able to fill out the questionnaire. This study targeted current internet users because e Health literacy can only be meaningfully assessed among people who have access to and use the internet.

### Exclusion criteria

Illiterate PHC attendees, those not using internet and those who refused to participate in the current study.

### Sample size

was calculated using OpenEpi program [30] **.**The pilot study revealed that 63% (13 out of 20) of attendants have sufficient E health literacy. With 0.05 confidence level, 0.05 precision and study power 80% then sample size is 359. This multiplied by 1.5 to compensate for sampling stratification. Thus the final sample was adjusted to be 538 participants, but a total of.

532 completed the questionnaire (response rate = 98.9%).

### Sampling method

**Fig. 1:**
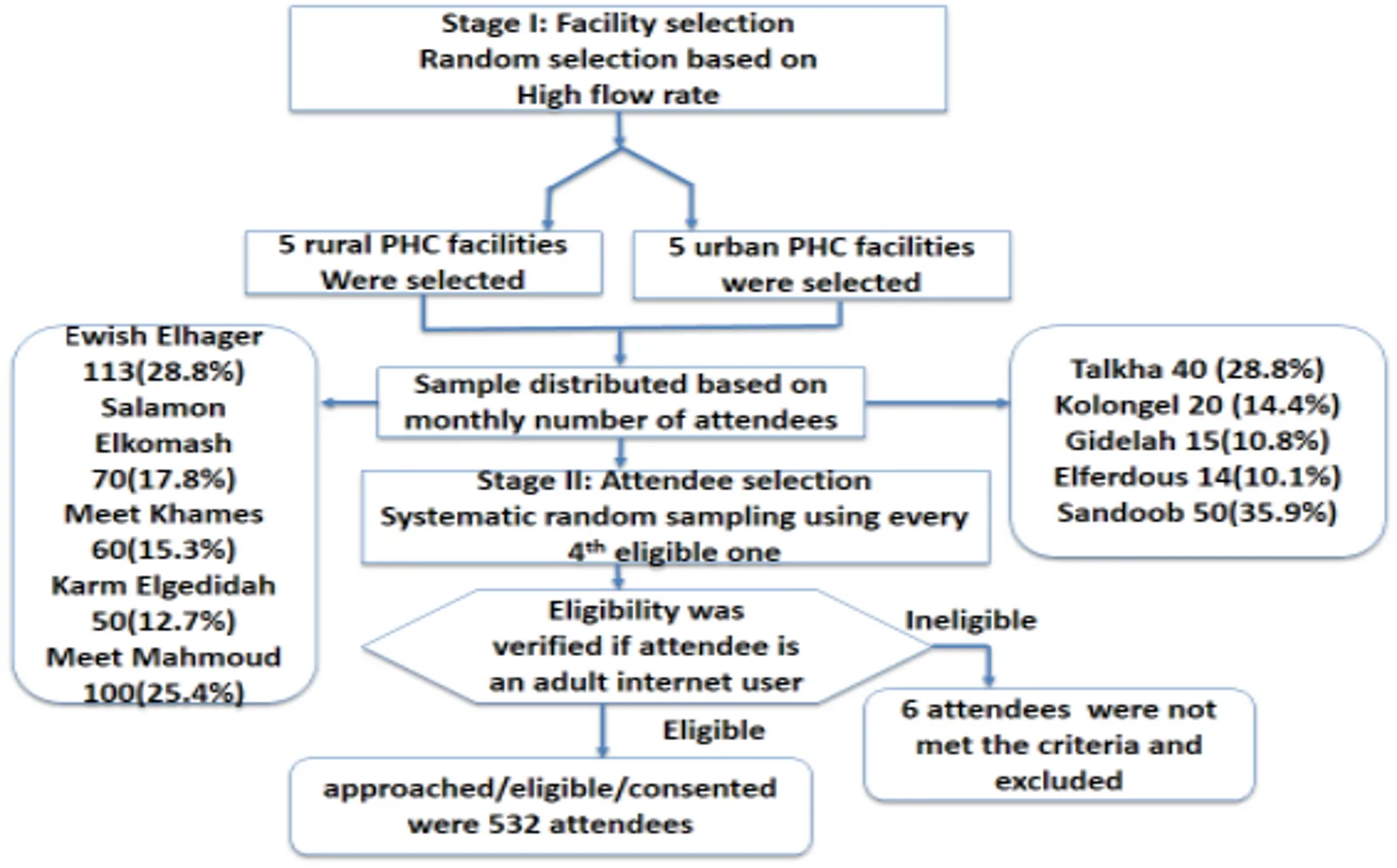
Flow diagram showing the selection process, recruitment flow and sampling methods

A multistage sampling method was used. Figure 1.

Stage I: PHC facilities were randomly selected, based on their high flow rate. Then, five urban settings namely (Talkha, Kolongel, Gidelah,& Elferdous, and Sandoob), and five rural facilities namely (Ewish Elhager, Salamon Elkomash, Meet Khames, Karm Elgedidah, and Meet Mahmoud) were selected. Sample was distributed proportionally between facilities according to monthly number of attendees.

Stage II: Participants were selected by systematic random sample. The first attendee was selected randomly, after which every 4th eligible attendee was invited to participate until the required sample size was completed. To be eligible to participate, participants had to be adult internet users.

All eligible attendees were approached, consent was obtained and the number approached, consented and included was documented achieving 98.9% response rate.

#### Data collection methodology

The data were collected by the researcher using structured validated Arabic questionnaire. The researcher explained the aim of the current study. The data were collected from 10 to 15 min after giving the time to read and fill out the questionnaire. The data were obtained under routine situations. The researcher was taught to verify the completeness and accuracy of data.

#### Study tools

An Arabic validated structured self-administered questionnaire (Appendix I) included the following sections:

First section: includes data about socio-demographic characteristics (age, sex, residence, education, employment, marital status, income, long term medication use, having medical diseases, self-rated health status, amount of general knowledge in health field, visiting doctor in the last 12 months, hospitalization in the last 12 months & reasons of PHC facilities attendance) after reviewing the previous literatures [25, 31].

Second section: includes data regarding internet characteristics (seeking of online health information, types of health information sought, reasons for seeking online health information, hours spent online per day, autonomy in accessing the internet, medical application use on own device, perceived usefulness and perceived importance of internet as a source of health information ( assessed on 5 point Likert scale (not usefulness at all / very useful ; not important at all /very important) retrieved from previous literature [6, 32] reliability of online health information ( assessed on 5 point Likert scale (very little/very much) was retrieved from previous literature [31] & devices used for online health information searching).

Third section: includes data about Arabic e health literacy Scale (Ar-eHEALS). It was originally developed by Norman and Skinner [33] and validated in Arabic by Wångdahl et al. [34]. The tool includes 8 items which assesses an individual’s perception of their skills and knowledge about finding, appraising, and using information online. Principal component factor analysis revealed a one-factor structure. Internal consistency was high (Cronbach’s α = 0.92); test-retest reliability was acceptable (weighted quadratic Cohen κ = 0.76), confirming its validity and reliability for measuring eHealth literacy among general population. The items were evaluated on 5 point Likert scale (1(strongly disagree) to 5 (strongly agree). Total scores range from 8 to 40, higher scores indicate higher e Health literacy. As no universally cutoff point for this scale, we adapted the cutoff point of 26 based on a previous validated Arabic tool conducted by Wångdahl et al. [34]. In addition, previous research adopted this cutoff point [23, 35]. Therefore, instead of imposing a sample-dependent threshold, we kept the previously proposed Arabic cutoff to maintain methodological consistency and allow comparison with the available literature. The tool was previously pilot tested among 20 persons who visited the targeted PHC setting and the reliability of the tool was tested using the Cronbach’s alpha which was 0.87, yielding satisfactory result. Those who included in the pilot study were excluded from the final analysis. While the Cronbach’s alpha of all sample was 0.90 indicating higher internal consistency of the tool in Egyptian context.The item-total correlation ranged from 0.59–0.71.The tool proved its structural validity among the study participants (Supplementary File II).

Fourth section: includes data about the perceived barriers of e Health literacy which retrieved from previous validated literatures [36, 37] e.g. ( low basic health literacy, limited skills in technology use, fear from technology use, Poor functionality of digital services, non-engaging, repetitive and lengthy content, Limited recognition of cultural concerns, Distrust of web health information, language barriers, lower privacy and confidentiality, lower standard of care, Lack of interaction with health care providers & Psychological barriers). After seeking the opinions of experts for relevance and clarity. The questions were formatted as yes/no allowing for multiple responses.

Statistical analysis: the collected data were collected, coded, processed and analyzed using IBM SPSS program version 26 (Armonk, NY: IBM Corp). No missing data were observed. Quantitative data were presented as mean and standard deviation (SD) .Qualitative variables were presented as number and percent. The normality of the data were assessed using the Shapiro-Wilk test. The chi-square test was employed to compare qualitative data. Before conducting the binary logistic regression analysis, multicollinearity among the independent variables were assessed using variance inflation factor (VIF) and tolerance values. Significant variables with *p* < 0.05 detected from bivariate analysis were entered simultaneously into a multiple logistic regression model using the Enter method to identify the independent factors of adequate e Health literacy. Crude odds ratio (COR), followed by adjusted odds ratio (AOR) and their 95% confidence interval (CI) were measured. *P* < 0.05 was considered statistically significant.

## Results

Out of 532 study participants were included in the final analysis, the majority aged (18–20) years (51.5%) with a mean age and standard deviation (SD) of (24.6 ± 8.9) years. Three quarter of them (75%) were female. Most of them resided in rural areas (73.9%), had university and postgraduate education (56.6%), current workers (51.3%), self-rated their health as good (39.7%), and accompanying their relative patients during visiting PHC settings (47.6%) (Table 1).

**Table 1:** Socio-demographic characteristics among studied participants during the study period (*n* = 532)

Variables	*N* (%)
Age (Mean ± SD), Median (Min-Max)	(24.6 ± 8.9), 20 (18–65)
18–20	274 (51.5)
21–30	196 (36.8)
> 30	62 (11.7)
Sex	
Male	133 (25)
Female	399 (75)
Marital status	
Single	366 (68.8)
Married	153 (28.8)
Divorced	7 (1.3)
Widowed	6(1.1)
Residence	
Rural	393 (73.9)
Urban	139 (26.1)
Education	
< secondary	2 (0.4)
Secondary	229 (43)
University &postgraduate	301 (56.6)
Occupation	
Current working (governmental& self-working)	273 (51.3)
Not working (housewives, retired, students)	259 (48.7)
Income Not enough	68 (12.8)
Enough for basic needs only	321 (60.3)
Enough for basic needs &luxuries	143 (26.9)
Having medical disease (Yes)^*^	136 (25.6)
DM	21 (3.9)
HTN	22 (4.1)
Cardiac disease	13 (2.4)
Hypercholestremia	15 (2.8)
Thyroid disease	14 (2.6)
Osteoarthritis	36 (6.8)
Asthma	32 (6)
Cancer	2 (0.4)
Depression	27 (5.1)
Long term medications (Yes)	91 (17.1)
Visited physician the last year(yes)	380 (71.4)
Hospitalized within the last year (yes)	51 (9.6)
Self-rated health status	
Very poor	16 (3)
Poor	21 (3.9)
Fair	159 (29.9)
Good	211 (39.7)
excellent	125 (23.5)
The amount of general knowledge in health field	
Very little	16 (3)
Little	53 (10)
To some extent	272 (51.1)
Much	118 (22.2)
Very much	73 (13.7)
Reasons of attending PHC facilities^*^	
Accompanying patient	253 (47.6)
Preventive assessment	196 (36.8)
Chronic disease treatment	61 (11.5)
Acute disease treatment	217 (40.8)
Request referral to a hospital	35 (6.6)

*Multiple answers were allowed; showing not mutually exclusive categories

Regarding internet use among study participants, the majority of them (91%) searched the internet for online health information. More than half of them (63%) used the internet to find more information. The most common type of health information searched on the internet was for a disease or health problem (50.9%). The majority (96.6%) of them used smartphones for online search. Third of them (31.6%) perceived internet usefulness as useful, however the minority of them (5.6%) had very much reliability about online health information (Table 2).

**Table 2:** Internet use characteristics among studied participants (*n* = 532)

Variables	*N* (%)
Searching internet for online health information yes No	484(91) 48(9)
If yes^*^, reasons of seeking online health information Gain more knowledge Manage my health more effectively Clarify information provided by a physician Find more information Out of interest and curiosity Look for alternatives or additional treatment options Verify the information discussed with a physician after the visit Insufficient information from a physician during the consultation Limited time with a physician during the consultation Disagree with a physician’s advice	342 (70.6) 198 (40.9) 90 (18.5) 305 (63) 197 (40.7) 51 (10.5) 28 (5.7) 8 (1.6) 5 (1) 1 (0.2)
Types of health related information sought^*^ A disease or health problem Drugs / medications Nutrition/diet/nutritional supplements Sports/aerobics/ physical exercise A specific doctor or a hospital Treatment/procedures Online support groups	271 (50.9) 166 (31.2) 239 (44.9) 155 (29.1) 40 (7.5) 228 (42.9) 27 (5.1)
Autonomy in accessing the internet No need for help Need help from family and friends	419 (78.8) 113 (21.2)
Hours spent weekly on the internet (Median) (MIN-MAX) 0.5- 2 3–5 6–10 > 10	6 (0.5–24) 68 (12.8) 14 5(27.3) 200 (37.6) 119 (22.4)
Using any medical application on your device Yes No	174 (32.7) 358 (67.3)
Using devices for online searching Smart mobile phones^*^ Tablet Laptop Desktop Others (smart watch, Smart TV)	514 (96.6) 104 (19.5) 81 (15.2) 28 (5.3) 12 (2.3)
Perceived internet usefulness Not useful at all Not useful Unsure Useful Very useful	21 (3.9) 50 (9.4) 80 (15) 168 (31.6) 213 (40)
Perceived internet importance Not important at all Not important Unsure important Very important	17 (3.2) 16 (3) 94 (17.7) 179 (33.6) 226 (42.5)
Reliability of online health information Very little Little To some extent Much Very much	19 (3.6) 87 (16.4) 305 (57.3) 91 (17.1) 30 (5.6)

*MIN* Minimum, *MAX *Maximum; hours groups were based on median

^*^Percentage based on multiple answers

Table 3 shows that mean (± SD) of E health literacy was 27.7 ± 5.3. Regarding the cutoff point used, more than two thirds (67.3%) had adequate e health literacy versus 32.7% of them had limited one. Female participants (*p* = 0.014), urban residents (*p* = 0.047), those who had excellent health (*p* < 0.001), those who had very much general knowledge in health field (*p* < 0.001), used the internet for health purposes (*p* < 0.001), those who had higher autonomy in accessing the internet (*p* = 0.001), those who spent more than 10 h on the internet per week (*p* = 0.01), those who perceived internet usefulness and importance (*p* < 0.001), and those who had high reliability about online health information (*p* = 0.015) had significantly adequate e health literacy compared with their counterparts.

**Table 3:** Distribution of e Health literacy among studied participants according to socio-demographic and internet use characteristics

Variables		e Health literacy (mean score ± SD) (27.7 ± 5.3)		Test of significance
Overall	532	Limited 174(32.7)	Adequate358 (67.3)	
I. Socio-demographic factors				
Age 18–20 21–30 > 30	274 196 62	92(33.6) 58(29.6) 24(38.7)	182(66.4) 138(70.4) 38(61.3)	χ^2^ = 1.9, *p* = 0.373
Sex Male Female	133 399	55(41.4) 119(29.8)	78(58.6) 280(70.2)	χ2 = 6.02, *p* = 0.014^*^
Marital status Single Married Divorced/Widowed	366 153 13	126(34.4) 42(27.5) 6(46.2)	240(65.6) 111(72.5) 7(53.8)	χ2 = 3.48, *p* = 0.176
Residence Rural Urban	393 139	138(35.1) 36(25.9)	255(64.9) 103(74.1)	χ2 = 3.9, *p* = 0.047^*^
Education < secondary Secondary University &postgraduate	2 229 301	1(50) 79(34.5) 94(31.2)	1 (50) 150(65.5) 207(68.8)	χ2 = 0.90, *p* = 0.636
Occupation Current working Not working	273 259	87(31.9) 87(33.6)	186(68.1) 172(66.4)	χ2 = 0.17, *p* = 0.672
Income Not enough Enough for basic needs only Enough for basic needs &luxuries	68 321 143	28(41.2) 102(31.8) 44(30.8)	40(58.8) 219(68.2) 99(69.2)	χ2 = 2.58, *p* = 0.274
Having medical disease Yes No	136 396	41(30.1) 133(33.6)	95(69.9) 263(66.4)	χ2 = 0.54, *p* = 0.461
Long term medications Yes No	91 441	28(30.8) 146(33.1)	63(69.2) 295(66.9)	χ2 = 0.18, *p* = 0.665
Visited physician in the last year yes No	380 152	123(32.4) 51(33.6)	257(67.6) 101(66.4)	χ2 = 0.07, *p* = 0.793
Hospitalized in the last year yes No	51 481	18(35.3) 156(32.4)	33(64.7) 325(67.6)	χ2 = 0.17, *p* = 0.679
Self-rated health status Very poor Poor Fair Good Excellent	16 21 159 211 125	10(62.5) 13(61.9) 61(38.4) 71(33.6) 19(15.2)	6(37.5) 8(38.1) 98(61.6) 140(66.4) 106(84.8)	χ2 = 34.4, *p* < 0.001^*^
The amount of general knowledge in health field Very little Little To some extent Much Very much	16 53 272 118 73	9(56.3) 24(45.3) 108(39.7) 25(21.2) 8(11)	7(43.8) 29(54.7) 164(60.3) 93(78.8) 65(89)	χ2 = 36.7, *p* < 0.001^*^
II. Internet use factors				
Searching internet for online health information yes No	484 48	146(30.2) 28(58.3)	338(69.8) 20(41.7)	χ2 = 15.7, *p* < 0.001^*^
Autonomy in accessing the internet No need for help Need help from family and friends	419 113	122(29.1) 52(46)	297(70.9) 61(54)	χ2 = 11.5, *p* = 0.001^*^
Hours spent weekly on the internet 0.5- 2 3–5 6–10 > 10	68 145 200 119	34(50) 47(32.4) 59(29.5) 34(28.6)	34(50) 98(67.6) 141(70.5) 85(71.4)	χ2 = 11.1, *p* = 0.01^*^
Using any medical application on your device Yes No	174 358	42(24.1) 132(36.9)	132(75.9) 226(63.1)	χ2 = 8.6, *p* = 0.003^*^
Perceived internet usefulness Not useful at all Not useful Unsure Useful Very useful	21 50 80 168 213	14(66.7) 32(64) 56(70) 53(31.5) 19(8.9)	7(33.3) 18(36) 24(30) 115(68.5) 194(91.1)	χ2 = 138.6, *p* < 0.001^*^
Perceived internet importance Not important at all Not important Unsure important Very important	17 16 94 179 226	13(76.5) 12(75) 63(67) 60(33.5) 26(11.5)	4(23.5) 4(25) 31(33) 119(66.5) 200(88.5)	χ2 = 124.3, *p* < 0.001^*^
Reliability of online health information Very little Little To some extent Much Very much	19 87 305 91 30	10(52.6) 33(37.9) 102(33.4) 26(28.6) 3(10)	9(47.4) 54(62.1) 203(66.6) 65(71.4) 27(90)	χ2 = 12.3, *p* = 0.015^*^

^*^Significant

Table 4 shows that tolerance values ranged from (0.82–0.98) for all the independent variables and the range of VIF values was from (1-1.2). As the conventional threshold of 5 and tolerance values were substantially above 0.10, suggesting no significant multicollinearity. Thus, the assumption of no multicollinearity was met and logistic regression analysis could proceed. Multiple logistic regression analysis shows that female participants (AOR = 1.8, 95% CI (1.1-3), *P* = 0.028), perceived internet usefulness (AOR = 5.6, 95% CI (2.9–10.7), *P* < 0.001), perceived internet importance (AOR = 5, 95% CI(1.8–13.3), *P* = 0.001), self- rated their health status as good and excellent (AOR = 3.7, 95% CI (1.6–8.9), *P* = 0.002), and fair (AOR = 2.7, 95% CI (1.1–6.7), *P* = 0.03), and having higher general knowledge in health field (AOR = 2.9, 95% CI(1.4–5.9), *P* = 0.004) independently associated with adequate e health literacy among study participants.

**Table 4:** Univariate and Multiple logistic regression analysis of independent factors of adequate e Health literacy among the studied participants

Independent factors	COR 95%CI	β	*p*	AOR, 95%CI	VIF	Tolerance
Sex Male Female	1(r) 1.6(1.1–2.5)	- 0.58	0.028^*^	1(r) 1.8(1.1- 3)	1	0.97
Residence Rural Urban	1(r) 1.5(1-2.3)	- 0.23	0.404	1(r) 1.2(0.7–2.2)	1	0.98
Perceived internet usefulness Not useful at all/ Not useful Unsure Useful/Very useful	1(r) 0.7(0.3–1.5) 7.8(4.5–13.6)	- 0.24 1.7	0.565	1(r) 1.2(0.5–2.8) 5.6(2.9–10.7)	1.2	0.83
Perceived internet importance Not important at all/ Not important Unsure Important/Very important	1(r) 1.5(0.6–3.8) 11.5(5.04–26.6)	-- 0.23 1.6	0.676 0.001^*^	1(r) 1.3(0.4–3.7) 5 (1.8–13.3)	1.2	0.82
Self-rated health status Very poor/Poor Fair Good/ Excellent	1(r) 2.6(1.3–5.5) 4.4(2.2–9.1)	- 1 1.3	0.03^*^ 0.002^*^	1(r) 2.7(1.1–6.7) 3.7(1.6–8.9)	1.1	0.93
The amount of general knowledge in health field Very little/ Little To some extent Much/ Very much	1(r) 1.3(0.8–2.3) 4.3(2.4-8)	- 0.28 1.1	0.391 0.004^*^	1(r) 1.3(0.6–2.5) 2.9(1.4–5.9)	1.1	0.94
Searching internet for online health information Yes No	3.2(1.7–5.9) 1(r)	0.25 -	0.511	1.2(0.5–2.8) 1(r)	1.1	0.87
Autonomy in accessing the internet No need for help Need help from family and friends	2.1(1.3–3.2) 1(r)	0.43 -	0.113	1.5(0.9–2.6) 1(r)	1.1	0.95
Hours spent weekly on the internet 0.5- 2 3–5 6–10 > 10	1 (r) 2.1(1.2–3.7) 2.3(1.3–4.2) 2.5(1.3–4.7)	- 0.29 0.56 0.55	0.429 0.122 0.158	1 (r) 1.3(0.6–2.7) 1.7(0.8–3.5) 1.7(0.8–3.7)	1	0.98
Using any medical application on your device Yes No	1.8(1.2–2.7) 1 (r)	0.29 -	0.263	1.3(0.8–2.2) 1 (r)	1	0.94
Reliability of online health information Very little& little To some extent Much& very much	1 (r) 1.3(0.8–2.1) 2.2(1.2–3.8)	- 0.19 0.44	0.536 0.234	1 (r) 0.8(0.4–1.5) 1.5(0.7–3.2)	1	0.98
% correctly predicted Model summary: Cox& Snell R square = 0.308 Nagelkerke R square = 0.429 Hosmer lemeshow test (χ2 = 3.952,*p* = 0.861)		78.4% -2 log likelihood = 477.334,*p* < 0.001				

*COR* Crude odds ratio, *AOR* Adjusted odds ratio, *CI* Confidence Interval, *VIF* Variance Inflation factor, *r* reference group

*Significant

The most common perceived barriers of electronic health literacy among study participants were low basic literacy (79.7%), lack of interaction with health care providers (73.3%), lack of recognition of cultural concerns (72.9%), presence of repetitive large online content (72.4%), limited skills to use technology (71.8%), poor functionality of digital services (69.5%), low standard of care (69.5%), and distrust of web health information (69%) (Table 5).

**Table 5:** Perceived barriers of e Health literacy among the studied participants (*n* = 532)

Barriers	*N* (%)
Low basic (functional) literacy*	424(79.7)
Limited skills to use technology	381(71.8)
Fear of technology	338(63.5)
Poor functionality of digital services (accessibility and cost of internet)	370(69.5)
Presence of repetitive large online content	385(72.4)
Distrust of web health information	367(69)
Psychological aspects related to online searching (fatigue, distress)	313(58.8)
Perceived lower privacy and confidentiality	335(63)
Lack of interaction with health care providers	390(73.3)
Low standard of care	370(69.5)
Cultural concerns recognition lack of	388(72.9)
Language constraints (preference of own language)	356(66.9)

*The literacy and numeracy skills required to read and understand health information

## Discussion

The current study aimed to assess the level of e Health literacy, its associated factors and explored the perceived barriers among internet using PHC attendees. The current study found that the mean score of e Health literacy was (27.7). This finding was similar to previous findings from South Korea (27.18) [38], and Malaysia (27.38) [39]. The possible explanation could be that the majority of our study included younger adults. This could also be related to the fact that younger adults have more internet penetration, interpreted technology proficiency than the older adult population which improve their e Health literacy. On the contrary, such a finding was higher than reported from previous research conducted among PHC patients in Malaysia (24.4) [25], Peru (18.2) [26] but lower than reported in previous international cross-sectional research (29.2) [23]. Differences in age, educational level, internet use patterns, and assessment tools among participants may explain their differences.

When considering the median cutoff point as reported in previous literatures [23, 34, 35], the current study found that more than two- thirds of our sample had adequate e Health literacy which agrees with prior studies conducted among internet users in Lebanon [9], Jordon [24] and Kuwait [13]. On the contrary, a previous research [27] conducted in Peru among PHC users which showed that half of participants had elementary and low level of e Health literacy while only one fifth of them had high level. Such a finding indicates that e Health literacy is slightly increasing in middle income nations showing that internet access and digital tools are increasingly required in everyday life. High rate of adequate e Health literacy is likely heavily inflated by this specific “young, educated, female accompanier” demographic among PHC internet users. This significantly limits the generalizability of findings to actual, older PHC patients. In addition, the lack of universally standardized cutoff point to assess the e Health literacy needs further investigation.

In the present study, female participants were independently associated with adequate e Health literacy which aligns with previous studies conducted in Peru [27], Lebanon [9], Kuwait [13], Jordon [24], and South Korea [38], Considering that women are more likely to seek medical attention and perceive better quality online health information [40]. However, this finding contradicts a previous Malaysian study conducted among PHC patients [25] which found no significant association between sex and e Health literacy. Although females had significantly adequate e Health literacy, such finding should be interpreted cautiously as females represented three-quarters of the study group. In addition, urban participants had significantly adequate e Health literacy than rural ones in the bivariate analysis. However, after adjusting for potential confounders in multiple logistic regression analysis, residence didn’t remain significant. This finding is in line with previous research [41, 42] which could be explained by the increased exposure to digital tools or reliable internet access among urban residents, which fosters earlier development of eHealth literacy skills [42]. This finding highlights the importance of equitable access to digital resources in enhancing e Health literacy especially in underserved communities [43]. Notably, many socio-demographic factors e.g. (age, employment, education, and income) were not significantly associated with adequate e Health literacy. However, these factors were significant factors e Health literacy as reported in previous research [10, 23, 25–27, 44, 45]. Such a finding could be explained by the inclusion of internet users who may be more affected by electronic engagement and confidence in using online health information than by socio-demographic characteristics alone. Therefore, national policies should target individuals with lower socioeconomic status among internet users to reduce health inequalities and improve their digital health literacy.

Consistent with e Health literacy Lily model [46] and prior studies, seeking the internet for online health information [22, 25, 47, 48], spending more time on internet weekly [46], and utilizing medical applications [41, 49, 50] were significantly associated with e Health literacy in the bivariate analysis. This can be attributed to the experiences they gained while seeking information, which refined their skills in assessing and utilizing that knowledge to make health-related decisions. However, these variables were not remained significant after adjustment in the regression model, this result may reflect broader underlying factors rather than independent ones of e Health literacy. Thus, PHC providers should be trained and provide awareness programs focusing on improving digital skills and empowering equitable access to reliable health information for those with limited e Health literacy.

Interestingly, participants who had high general health knowledge were independently significantly associated with adequate e Health literacy. This finding agrees with a previous study [31] conducted among primary school teachers which found that having higher general health knowledge significantly associated with the e Health literacy. This finding highlights the reciprocal influence of e Health literacy and health behavior of individuals. The study also showed that the majority of people who experienced adequate e Health literacy had the ability to evaluate and recognize the correctness of health-related information and use them in their daily lives. Similarly, previous research [31, 51, 52] supports such finding. However, a previous Iranian study [53] contradicts our results. Consistent with WHO global strategy on digital health (2020–2027) [54], national policies should focus on how trusted online content could improve digital health literacy among those with limited one.

Rating one’s health as good or excellent was associated with higher motivation to use electronic Health. In the current study, those who were self-rated their health as good or excellent significantly were significant independent factor of adequate e Health literacy. This finding aligns with by previous studies [23, 39, 55]. Interestingly, the current study found that participants who had higher autonomy in accessing the internet were significantly associated with adequate e Health literacy compared to their counterparts which aligns with a previous finding among PHC users in Peru [27].

The strong observed association between those perceived internet usefulness, importance and e Health literacy aligns with previous studies conducted in Kuwait [13], and Lebanon [9]. Such association could be explained by the Technology Acceptance Model (TAM) [56], which posits that individuals are more inclined to engage with digital health technologies when they believe them to be beneficial. A previous research conducted among Swedish PHC visitors showed that those perceived the internet as not useful had lower ehealth literacy [57] explaining the mutual effect of individuals’ intention to use information technology (IT) and their e Health literacy. These results confirm the theoretical framework of the TAM, demonstrating that perceived internet usefulness and importance of the Internet are key factors of e Health literacy. Thus, national strategies should focus on the practical value and use of digital health resources in a useful manner to improve the public engagement.

Despite the high prevalence of adequate e Health literacy, significant proportion of participants had limited one. Therefore, to bridge this digital divide, the present study explored numerous perceived barriers of digital health literacy. The majority of our participants reported that low basic literacy was the common barrier of e Health literacy. Such finding is consistent with a previous research [37]. Therefore, health education messages should be simplified by the simplification of language. Furthermore, the presentation of knowledge by way of a multiplicity of forms (audio, video, and text) can potentially enable people to grasp basic messages without the barrier of language or literacy [58].

Another important barrier of the current study was the limited skills to use technology and fear of it which is consistent with a previous review [59] suggesting several mitigation strategies for culturally diverse populations. Therefore, training programs should be applied within PHC services to encourage the technology use, clarify the methods of searching medical information and making healthy decision. In the current study poor functionality of digital services regarding accessibility and cost of internet was also perceived a common barrier. Previous research confirmed such finding [60]. Improving access to free internet in public spaces (e.g., community libraries) may also be a useful strategy. This access should address concerns about privacy noted in a previous study [61] and also the irregular availability of public internet services [17].

In line with our study, a previous research [62] reported lack of interaction with health care providers was a key barrier of the e Health literacy. In addition, lack of recognition of cultural concerns was reported as a barrier to the use of digital health tools (DHTs) in a previous research [63]. Thus, ensuring the content and approach are relevant to the cultural community such as using stories that reflect their experience, or ensuring that any health care providers are of the same cultural background is a vital issue to bridge this digital health divide. Web-based information distrust was another theme. Similarly, Hyman et al. [64] found that web-based information, especially its currency and reliability, was distrusted. Some comparative studies suggest that cultural theories may explain differences in trust between populations (e.g., individual vs. collective societies), while others suggest that mistrust of the medical system may also affect attitudes toward web-based health information [65, 66]. Further research should examine whether linguistically and culturally appropriate DHTs are trusted and whether community leaders championing their use may improve cultural relevance.

The current study is consistent with prior research which reported that psychological aspects (e.g. distress and fatigue) [63], loss of privacy [62], and low standard of care including low opportunity of physical assessments [67] were among the barriers of e Health literacy. Therefore, future research will be recommended to uncover these barriers and finding appropriate solutions, as a way to bridge the digital divide in health across different populations.

In light of these findings, the current study provides insight into implementing future interventions including routine assessment of e Health literacy at PHC visits, training of PHC providers to guide individuals to reliable online resources and development of culturally appropriate Arabic digital health materials tailored to patients with low e Health literacy. These strategies align with national digital health policy (2025–2029) and WHO global strategy on digital health and have the potential to improve e Health literacy and reduce digital health inequalities in Egypt.

### Strengths and limitations of the study

To our knowledge, this is the first Egyptian study addressing e Health literacy and its associated factors among internet users of PHC attendees including a large sample size. It also explored perceived barriers to digital health literacy. The study also validated the tool in Egyptian context. In addition, the inclusion of rural and urban facilities improves the representation of participants within the targeted facilities. However, it has some limitations. First, the cross-sectional designs do not allow causal associations between e Health literacy and its associated factors. Secondly, use of self- reported data might lead to recall as well as social desirability bias. Third, the predominance of younger ages, females and the exclusion of illiterate individuals and non-internet users limits the generalizability of the findings, particularly to populations at greatest risk of digital exclusion. Fourth, absence of universally standardized cutoff point of the scale could limit the comparison across studies. Fifth, inability to implement multilevel modeling because of presence of limited numbers of facilities and will be considered in future multicenter studies. Also, a number of bivariate comparisons were performed, but interpretation was primarily based on the adjusted multivariable analyses.

## Conclusions

This study revealed that around two-thirds of internet using PHC attendees had adequate e Health literacy. Adequate e Health literacy was independently associated with being female, having better self-rated health, higher general health knowledge, and perceiving greater usefulness and importance of the internet for health-related purposes. Low basic literacy, limited interaction with health care providers, and lack of consideration of cultural concerns were the most common barriers to e Health literacy. These findings highlight areas that may guide future interventions to improve e Health literacy in PHC settings including improving digital health education, promoting the use of reliable online health resources, enhancing healthcare provider engagement and developing culturally appropriate digital health materials. However, the results should be interpreted with caution, because of the cross-sectional design, the use of self-reported data and the fact that the sample was predominantly composed of young, female, educated from one district. These characteristics may limit the generalizability of the results to the broader Egyptian PHC population and do not allow causal inferences. Further multicenter longitudinal studies with more representative populations, including older adults and those with limited digital access, are needed to confirm these findings and further explore factors associated with e Health literacy. And the urgent need for qualitative work to deepen understanding of barriers.

## Supplementary Information

Supplementary Material 1.

Supplementary Material 2.

## Data Availability

Data are available upon request from the corresponding author.

## Abbreviations

AOR: Adjusted odds ratio
Ar-eHEALS: Arabic e health literacy Scale
CI: Confidence Interval
COR: Crude odds ratio
COVID-19: Corona virus disease-19
DHTs: Digital health tools
ICT: Information communication technology
IRB: Institutional Research Board
LMICs: Low-middle income countries
PHC: Primary health care
SD: Standard deviation
SDGs: Sustainable developmental goals
SPSS: Statistical Packages for the Social Sciences
VIF: Variance inflation factor
WHO: World Health Organization
