# Supplementary material for: Assessment of electronic health literacy and its associated factors among internet -using primary health care attendees in Mansoura district, Egypt: a cross-sectional study
Source: BMC Public Health. 2026 Aug 19;26:2398. doi: 10.1186/s12889-026-28907-8 (PMC13491989; doi:10.1186/s12889-026-28907-8)
Supplement: Supplementary file 1 [file 12889_2026_28907_MOESM1_ESM.docx]

**Appendix I**

**I: Socio-demographic characteristics: Part**

1. Age: ……………
2. Sex: Male	 Female
3. Marital status:  Single	Married Divorced Widowed
4. Educational level: Primary	 Preparatory	General Secondary / Technical Secondary	University student  University graduate	 Postgraduate (Master's / Doctorate)

5. Occupation: Government employee	Self-employed

Not working/Retired / Housewife	 university student
6. Income: Not enough	Enough for basic needs only	Enough for basic needs and luxuries
7. Residence: Rural Urban
8. Do you have any medical conditions? Yes	No	If yes (Multiple answers allowed)	 Diabetes	Hypertension	Cardiac disease	Cancer	Depression	Osteoarthritis Thyroid disease	 Hypercholestremia	Asthma
9. Do you use any long-term medications? Yes	No
10. What is your assessment for health status? Very poor	Poor	Fair	Good	 Excellent
11. What is your level of general knowledge in the health field?

Very little	Little	To some extent	Much	Very much
12. Did you visit the physician in the past year? Yes	 No
13. Have you been hospitalized in the past year? Yes	 No
14. Reasons for visiting primary healthcare facilities (Multiple answers allowed): Treatment of a chronic disease	 Treatment of an acute disease	 preventive assessment	Request for hospital referral	Accompanying a patient.

**Part II: Internet use characteristics:**

15. Are you Searching internet for online health information? Yes	No

If yes (answer the following questions)
16. Reasons for seeking online health information (select more than one answer):

- Gain more knowledge
- Manage my health more effectively
- Clarify information provided by a physician
- Find more information
- Out of interest and curiosity
- Look for alternatives or additional treatment options
- Verify the information discussed with a physician after the visit
- Insufficient information from a physician during the consultation
- Limited time with a physician during the consultation
- Disagree with a physician's advice
  17. Types of health information you are looking for (multiple choices allowed):
  Disease or health issue
- Treatment or medical procedure
- Medications/Vaccinations
- Nutrition, diet, or dietary supplements
- Sports, aerobic exercises, and physical exercises
- Specific doctor or hospital
- Online support groups

18. Autonomy in accessing the internet:

No need for help	 Need help from family and friends

19. How many hours do you spend using the internet per week? .......

20. Do you use any medical applications? Yes No

21. What devices are used for internet searching?  Smart mobile phone laptop desktopcomputer tablet others (please specify).
22. Do you trust health-related information on social media?

Very little Little To some extent Much Very much

23. How useful do you feel the Internet is in helping you in making decisions about your health?

Not useful at all not useful unsure	 useful very useful
24. How important is it for you to be able to access health resources on the Internet?

Not important at all not important unsure Important Very important **Part III: eHealth Literacy Scale**

1. I know **what** health resources are available on the Internet

1) Strongly Disagree

2) Disagree

3) Undecided

4) Agree

5) Strongly Agree

2. I know **where** to find helpful health resources on the Internet

1) Strongly Disagree

2) Disagree

3) Undecided

4) Agree

5) Strongly Agree

3. I know **how** to find helpful health resources on the Internet

1) Strongly Disagree

2) Disagree

3) Undecided

4) Agree

5) Strongly Agree

4. I know **how to use** the Internet to answer my questions about health

1) Strongly Disagree

2) Disagree

3) Undecided

4) Agree

5) Strongly Agree

5. I know how to use **the health information** I find on the Internet to help me

1) Strongly Disagree

2) Disagree

3) Undecided

4) Agree

5) Strongly Agree

6. I have the skills I need to **evaluate** the health resources I find on the Internet

1) Strongly Disagree

2) Disagree

3) Undecided

4) Agree

5) Strongly Agree

7. I can tell **high quality** health resources from **low quality** health resources on the Internet

1) Strongly Disagree

2) Disagree

3) Undecided

4) Agree

5) Strongly Agree

8. I feel **confident** in using information from the Internet to make health decisions

1) Strongly Disagree

2) Disagree

3) Undecided

4) Agree

5) Strongly Agree

**Part IV: Perceived barriers of electronic health literacy: Answer the questions (Yes/ No): (Multiple choices allowed)**

Low basic literacy

Limited skills to use technology

Fear of technology

Poor functionality of digital services (accessibility and cost of internet)

Presence of repetitive large online content

Distrust of web health information

Psychological aspects related to online searching (fatigue, distress)

lower privacy and confidentiality

Lack of interaction with health care providers

Low standard of care

Lack of recognition of cultural concerns

Language constraints (preference of own language)

**Thank you**
