# Supplementary material for: Assessment of electronic health literacy and its associated factors among internet -using primary health care attendees in Mansoura district, Egypt: a cross-sectional study
Source: BMC Public Health. 2026 Aug 19;26:2398. doi: 10.1186/s12889-026-28907-8 (PMC13491989; doi:10.1186/s12889-026-28907-8)
Supplement: Supplementary file 2 [file 12889_2026_28907_MOESM2_ESM.docx]

**Factor structure of Ar-eHEALS among Egyptian sample**

| **Descriptive Statistics** | | | |
| --- | --- | --- | --- |
|  | Mean | Std. Deviation | Analysis N |
| Q1 | 3.27 | .858 | 532 |
| Q2 | 3.46 | .832 | 532 |
| Q3 | 3.53 | .826 | 532 |
| Q4 | 3.63 | .887 | 532 |
| Q5 | 3.61 | .837 | 532 |
| Q6 | 3.40 | .902 | 532 |
| Q7 | 3.41 | .886 | 532 |
| Q8 | 3.42 | .910 | 532 |

| **KMO and Bartlett's Test** | | |
| --- | --- | --- |
| Kaiser-Meyer-Olkin Measure of Sampling Adequacy. | | .916 |
| Bartlett's Test of Sphericity | Approx. Chi-Square | 2165.183 |
|  | df | 28 |
|  | Sig. | .000 |

| **Communalities** | | |
| --- | --- | --- |
|  | Initial | Extraction |
| Q1 | 1.000 | .473 |
| Q2 | 1.000 | .625 |
| Q3 | 1.000 | .616 |
| Q4 | 1.000 | .613 |
| Q5 | 1.000 | .611 |
| Q6 | 1.000 | .622 |
| Q7 | 1.000 | .553 |
| Q8 | 1.000 | .606 |
| Extraction Method: Principal Component Analysis. | | |

| **Total Variance Explained** | | | | | | |
| --- | --- | --- | --- | --- | --- | --- |
| Component | Initial Eigenvalues | | | Extraction Sums of Squared Loadings | | |
|  | Total | % of Variance | Cumulative % | Total | % of Variance | Cumulative % |
| 1 | 4.717 | 58.966 | 58.966 | 4.717 | 58.966 | 58.966 |
| 2 | .794 | 9.928 | 68.893 |  |  |  |
| 3 | .599 | 7.492 | 76.385 |  |  |  |
| 4 | .477 | 5.962 | 82.347 |  |  |  |
| 5 | .378 | 4.727 | 87.074 |  |  |  |
| 6 | .373 | 4.665 | 91.739 |  |  |  |
| 7 | .337 | 4.217 | 95.956 |  |  |  |
| 8 | .324 | 4.044 | 100.000 |  |  |  |
| Extraction Method: Principal Component Analysis. | | | | | | |

| **Component Matrix^a^** | |
| --- | --- |
|  | Component |
|  | 1 |
| Q1 | .688 |
| Q2 | .790 |
| Q3 | .785 |
| Q4 | .783 |
| Q5 | .782 |
| Q6 | .789 |
| Q7 | .744 |
| Q8 | .778 |
| Extraction Method: Principal Component Analysis. | |
| a. 1 components extracted. | |
